# Downregulation of PSCA promotes gastric cancer proliferation and is related to poor prognosis

**DOI:** 10.7150/jca.33575

**Published:** 2020-02-19

**Authors:** Li-pu Xu, Hai-bo Qiu, Shu-qiang Yuan, Yong-ming Chen, Zhi-wei Zhou, Ying-bo Chen

**Affiliations:** 1 State Key Laboratory of Oncology in South China, Sun Yat-sen University Cancer Center, Guangzhou, China; 2 Collaborative Innovation Center for Cancer Medicine, Sun Yat-sen University Cancer Center, Guangzhou, China; 3 Department of Gastric Surgery, Sun Yat-sen University Cancer Center, Guangzhou, China

**Keywords:** * PSCA*, gastric cancer, prognosis, survival, proliferation

## Abstract

**Background:** Dysregulation of prostate stem cell antigen (PSCA) has been implicated in human cancers. Studies have reported that PSCA expression is generally high in prostate cancer, which correlates with a worse survival. PSCA is also highly expressed in bladder, ovarian, and pancreatic cancers. However, PSCA is expressed at low levels in gastric, gallbladder and oesophageal cancers. At present, the clinical significance, expression pattern and biological function of PSCA in gastric cancer (GC) are still unclear.

**Methods:** Previously, we used cDNA microarray as a screening tool to compare GC tissues with its matched normal gastric mucosa tissues (MNGT), and obtained the differentially expressed genes of the two tissue types. PSCA is one of the genes significantly down-regulated in GC tissues. In this study, we detected the expression of PSCA in GC tissues and MNGT by western-blot experiment and immunohistochemical staining (IHC). Then the relationship between the expression pattern of PSCA and the clinicopathological characteristics and survival in GC was analyzed. In order to further study the function of PSCA in GC, lentivirus was used to construct stable cell lines with knockdown and overexpression of PSCA gene. We used AGS and MKN45 cell lines for plasmid transfection. Colony formation assay, MTS and nude mice xenograft model were performed to investigate the effect of PSCA in GC.

**Results:** Western-blot and IHC assays demonstrated that the expression of PSCA in GC tissues was significantly lower than that in the MNGT. PSCA expression in GC tissues was high in 252 (57.5%) and low in 186 (42.5%) of 438 patients. PSCA expression for MNGT was high in 273 (62.3%) and low in 165 (37.7%) of 438 patients. PSCA expression was significantly associated with T classification (P=0.024), N classification (P=0.018) and TNM stage (P=0.019) using χ^2^ test. The relationship between PSCA expression level and patient survival was analysed using Kaplan-Meier analysis and the log-rank test. Low levels of PSCA expression were significantly associated with a poorer OS than high expression levels of PSCA (P=0.011). In the COX regression analysis of OS, all 9 variables in the univariate analysis were significantly correlated with OS (P<0.05), while the variables found to be independently correlated with OS in the multivariate analysis were PSCA expression (P=0.036), age (P<0.001), gender (P=0.007), and TNM stage (P<0.001), respectively. Univariate and multivariate analyses showed that PSCA was an independent prognostic factor for OS in GC. *In vitro* MTS cell proliferation experiment and clonal formation experiment and *in vivo* nude mouse subcutaneous tumorigenesis experiment all proved that knockdown of PSCA gene can improve the proliferation ability of GC cells, while *in vitro* experiment proved that overexpression of PSCA can reduce the proliferation ability of GC cells.It was found that knockdown of PSCA gene can improve the proliferation ability of GC cells both *in vitro* and *in vivo*, while overexpression of PSCA can reduce the proliferation ability of GC cells *in vitro*.

**Conclusion:** Our study showed that the expression of PSCA gene was decreased in GC, which was related to more advanced pathological stages. And the expression level of PSCA in GC was an independent good prognostic factor. PSCA gene had the function of inhibiting GC proliferation.

## Introduction

Gastric cancer (GC) is the fifth most common cancer and was the third leading cause of cancer deaths worldwide in 2018.^1^ More than 1 million new GC cases were diagnosed in 2018, and more than 783 thousand deaths were attributed to GC.^1^ That is, approximately 1 in every 12 deaths was caused by GC globally.^1^ To date, surgical resection remains the primary treatment for GC. However, most patients are diagnosed with advanced or stage IV TNM GC.^2^ Operation alone has a limited effect on patient survival. Therefore, chemotherapy and targeted therapy are of great importance for GC treatment. To date, targeted drugs applied in GC are limited to ramucirumab and trastuzumab.^3^-^5^ More targeted drugs are required to be exploited and to be used in the clinical treatment of GC.

PSCA gene function is to encode a glycosylphosphatidylinositol-anchored cell membrane glycoprotein, which is related to cell adhesion, proliferation, and survival. PSCA is on chromosome 8q24.3 with a polymorphism that induced different risks for different cancers.^6^ Studies have reported that PSCA expression is generally high in prostate cancer,^7^ which correlates with a worse survival. PSCA is also highly expressed in bladder, ovarian, and pancreatic cancers.^8^-^10^ However, PSCA is expressed at low levels in gastric, gallbladder and oesophageal cancers.^10^, ^11^ These results indicate that PSCA has different functions in different tissue types. Initially, PSCA was considered to participate in signal transduction, and several studies have shown its involvement in cell growth regulation.^9^-^11^ Subsequently, studies have shown PSCA promotes prostate cancer growth by increasing c-myc expression through the PI3K/AKT signalling pathway 12. Nevertheless, a study of PSCA in esophageal cancer showed that PSCA plays a tumor suppressor role by promoting the nuclear translocation of RB1CC1.^13^ Recently, a genome-wide Association Study (GWAS) in Japan found that PSCA gene loci rs2294008, rs2976392 and other variants were associated with the incidence of gastric adenocarcinoma.^14^ However, the role of PSCA in GC is still unknown, and the function of PSCA in GC has not been fully explored.

Previously, we used cDNA microarrays as a screening tool; we compared GC tissues with their MNGT and obtained genes with differential expression in both tissue types. PSCA was one of the significantly downregulated genes in GC tissues. Therefore, in this study, we examined PSCA expression in GC tissues and MNGT, compared the clinicopathologic and prognostic differences between the high and low expression levels in PSCA patients, and investigated the function of PSCA in GC, laying the foundation for the next step to clarify the mechanism of PSCA function in GC.

## Materials and Methods

### Patients and tissue samples

All 438 pairs of paraffin-embedded GC tissues and their MNGT were obtained from surgical resection at Sun Yat-sen University Cancer Center and then made into tissue microarrays (TMAs). All the patients involved in this study signed informed consent agreements. The use of paraffin-embedded tissues in this study was approved by the Ethics Committee of Sun Yat-sen University Cancer Center. The patients were selected continuously retrospecttively from operation time during March 2010 to November 2013 in our department, included the conditions as follows:1) no history of neoadjuvant therapy, 2) underwent gastric resection, whatever operation kind (palliative or radical, partial or total gastric resection, D1,D2 or D3 lymphadenectomy, R0 or R1 resection), 3) no history of malignant tumour other than GC, 4) postoperative pathology confirmed gastric adenocarcinoma or squamous carcinoma. Exclusive criteria were: 1) no gastrectomy, and 2) R2 resection. Follow-ups were conducted by the Department of Follow-up in our hospital every 3 months after surgery for the first 2 years, every 6 months from the third to fifth years after surgery, and then once a year until patient death. The TNM stage was measured using The American Joint Committee on Cancer (AJCC, 8^th^ edition).

### Immunohistochemistry

Immunohistochemistry was performed on the abovementioned paraffin-embedded tissues. The tissues were fixed, paraffin-embedded and cut into 5 μm thick slices. IHC staining was performed according to the standard IHC protocol. First of all, one of the TMA slides were placed on the microscope which was connected to the computer. On the computer side set the diameter size of a circular tissue point in the TMA slide, adjusted the appropriate focal length. Next, the microscope took several photos for each tissue point of the TMA slide, then the computer jointed the photos together into a complete picture of the TMA slide and marked each tissue point of the TMA slide. The TMA IHC images were photographed by a TMA scanning system (Vectra 2.0.4982.22572, Perkin Elmer, Inc.). Then the computer analyzed each tissue point of the TMA slide by tissue scanning analysis system software provided with the instrument (Perkin Elmer, Inc.). All sells in a marked tissue point were evaluated by the computer software for staining intensity and then the percentages of each intensity grade were calculated. The staining intensity was defined as follows: 0 (negative), 1 (weak), 2 (moderate), and 3 (strong). The immunoreactive score (IRS) was multiplied by the grade of positive cell percentages and the staining intensity of the tissue. The final score of one TMA tissue was added by each staining intensity multiplied by its own percentage * 100. For example, if a TMA tissue staining intensity was 0 (18.92%), 1+ (80.61%), 2+ (0.47%) and 3+ (0.00%), then the final score was (0*18.92%+1*80.61%+2*0.47%+3*0.00%) * 100=82. The results of all TMA tissues ranged from 2 to 198. We defined the level of PSCA as low (score<78) and high (score ≥78) to make sure enough number of cases for both groups.

### Cell lines and cell culture

MKN45 and AGS cells were acquired from the Shanghai Cell Bank of the Chinese Academy of Sciences (Shanghai, China). MKN45 cells were cultured in RMPI-1640 media (Gibco) supplemented with 10% foetal bovine serum (Gibco). AGS cells were cultured in F-12K media (Gibco) supplemented with 10% foetal bovine serum (Gibco). MKN45 and AGS cells grew in standard cell culture conditions (37 °C, 5% CO_2_, and 95% humidity).

### Antibodies

Anti-PSCA rabbit polyclonal antibody was purchased from Abcam (ab64919, Abcam, Inc., Cambridge, MA). Anti-β-actin mouse monoclonal antibody, goat anti-rabbit conjugated to horseradish peroxidase secondary antibody, and goat anti-mouse conjugated to horseradish peroxidase secondary antibody were purchased from Cell Signaling Technology (CST).

### Western blot analysis

Total protein was extracted from GC tumour tissues and MNGT using lysis buffer. Tissues were obtained from surgery resection and confirmed by pathological diagnosis. Total protein was measured by a BCA kit and then made into protein samples. Protein samples were separated by sodium dodecyl sulphate polyacrylamide gel electrophoresis (SDS-PAGE) using a Bis-Tris gel and were transferred to PVDF membranes by wet transfer. The acrylamide concentration was 15%. The gel was run at 80 V for 30 min and 120 V for 65 min. Then, the gel was transferred to PVDF membranes at 0.25 A for 90 min. The PVDF membranes were blocked in 5% skim milk at 4 °C overnight and then incubated in anti-PSCA rabbit polyclonal antibody (1:1000 dilution) and anti-β-actin mouse monoclonal antibody (1:1000 dilution) at 4 °C overnight. The PVDF membranes were incubated in goat anti-rabbit conjugated to horseradish peroxidase secondary antibody (1:3000 dilution) and goat anti-mouse conjugated to horseradish peroxidase secondary antibody (1:5000 dilution) for 1 h. The membranes were visualized via ECL reagents (Bio-Rad, USA) and measured by the Bio-Rad instrument.

### MTS assay

The cell proliferation rate was measured by a 3-(4,5-dimethylthiazol-2-yl)-5-(3-carboxymethoxyphenyl)-2-(4-sulfophenyl)-2H-tetrazolium (MTS) assay (Promega Corporation, Fitchburg, WI). According to the instructions, 2000 cells were plated in wells of a 96-well plate. A total of 6 plates were used for 1-6 days. MTS (10 µl) was added to the wells and then incubated in a cell incubator for 4 h. The plates were measured at 490 nm wavelength in the machine.

### Colony formation assay

MKN45, MKN45-SH, AGS and AGS-NM cells were seeded in 6-well plates at 500 cells per well and cultured for 10 or 14 days. Cells were fixed with 4% paraformaldehyde solution, stained with 0.1% crystal violet, and counted under an inverted microscope.

### Slow virus and transfection

Plasmids, including knockdown or overexpresssion sequences, were designed and packaged as lentiviruses by GenePharma. The knockdown sequences were 5'-GCTGTGACACCGACTTGTGCA-3'. Transfection steps were performed according to GenePharma protocols. Selection was performed using puromycin for stable transfection for 14 days.

### Nude mice xenograft

The Research Animal Resource Center of Sun Yat-sen University approved the use of laboratory animals in this study. BALB/c athymic nude mice (4 weeks old) were purchased from Beijing Vital River Laboratory Animal Technology Co., Ltd. (Beijing, China). The experiments and procedures on animals conformed to the institutional animal care guidelines. A xenograft model was used. The mice were injected with 5 x 10^6^ MKN45 cells or MKN45-shPSCA cells in the right subcutaneous armpit. Tumour size was measured twice a week. Mice were sacrificed after the size of the tumour exceeded 1000 mm^3^.

## Results

### PSCA expression in GC patients and its relationship to clinicopathological characteristics

To investigate the relationship between PSCA expression and the clinicopathological features of GC patients, IHC was performed to evaluate the PSCA expression pattern of 438 pairs of paraffin-embedded GC tissues and their MNGT in TMA. The PSCA antibody specifically recognized PSCA in the cellular membrane and cytoplasm. PSCA expression in GC tissues was high in 252 (57.5%) and low in 186 (42.5%) of 438 patients. PSCA expression for MNGT was high in 273 (62.3%) and low in 165 (37.7%) of 438 patients. The difference in PSCA expression indicates that PSCA protein levels were lower in tumour tissues than in MNGT tissues (Table 1). Western blot analysis further confirmed these results (Figure A_a1, Figure A_a2). The general characteristics of patients is shown in Table 2. Typical PSCA staining intensity images (negative, weak, moderate and strong) from TMA tissues are presented in Figure A_b. As shown in Table 3, PSCA expression was significantly associated with T classification (P=0.024), N classification (P=0.018) and TNM stage (P=0.019). The relationship between PSCA expression level and patient survival was analysed using Kaplan-Meier analysis (Figure B) and the log-rank test. Low levels of PSCA expression were significantly associated with a poorer OS than high expression levels of PSCA (P=0.011). These results suggest that PSCA is related to GC tumourigenesis. The univariate and multivariate analysis of COX regression for overall survival (Table 4) suggested that PSCA expression was an independent prognostic factor for gastric cancer (P=0.036).

### PSCA decelerates GC cell proliferation *in vitro*

First, the PSCA expression level in various GC cell lines was assessed. The highest expression level of PSCA was in MKN45 cells, and the lowest expression level is AGS cells (Figure C_a). Therefore, MKN45 and AGS cells were selected to evaluate the function of PSCA in GC cells. Lentiviral vectors expressing PSCA and PSCA shRNA sequences were used to establish stable cell lines. MKN45-shPSCA and AGS-PSCA were then constructed by lentivector transduction. The WB assay confirmed that the silencing operation in MKN45 cells and the overexpression operation in AGS cells succeeded (Figure C_b, Figure C_c).

MTS assays were performed to evaluate cell proliferation. Downregulated PSCA levels in MKN45 cells significantly increased cell growth compared with MKN45 cells (Figure D_a, P=0.001). On the other hand, upregulated PSCA levels in AGS cells decreased cell growth compared to control cells (Figure D_a, P<0.001).

As shown in Figure D_b, the colony formation assay was in accordance with the observation. The number of colonies for MKN45-shPSCA was significantly higher than the numbers of the MKN45 control cells, and the AGS-PSCA cells formed more colonies than those of the AGS cells.

### PSCA decelerates GC cell proliferation *in vivo*

To further investigate the observation that PSCA suppresses GC cell proliferation. Nude mice xenograft tumour model assays were performed. Male nude mice were divided into two groups with five animals in each group. A total of 5 x 10^6^ of MKN45 or MKN45-shPSCA cells per mouse were injected into the right subcutaneous axillary in each group. As shown in Figure E_b, the tumour volumes were significantly higher in the MKN45-shPSCA group than in the MKN45 group at every specific measuring time. The mice were sacrificed using cervical dislocation after 4 weeks when the largest tumour volume reached 1000 mm^3^. The tumour was excised from each mouse, and the weight of the tumour was measured. Again, the tumour weights in the MKN45-shPSCA group were significantly greater than those in the MKN45 group (Figure E_a, P=0.037).

## Discussion

In our previous cDNA microarray screening, we found that PSCA was significantly expressed at low levels in GC. To further investigate the role of PSCA in GC, we performed western blot and IHC assays. A total of 438 pairs of GC tissues with its MNGT were selected to compare the expression difference of PSCA, and found that PSCA gene expression was significantly lower in GC tissues which was consistent with the study of Sakamoto et al.^11^ and the sequencing results in our department. A GWAS study predicted that low PSCA expression might be associated with more advanced clinical staging and poor prognosis.^15^ Our study found that low expression of PSCA was associated with higher pT staging (P=0.024), pN staging (P=0.018), and pTNM staging (P=0.019), especially in 15 of the 17 patients with pN3b. Kaplan-meier survival analysis showed that low PSCA expression was associated with poor prognosis (P=0.011), while univariate and multivariate analysis showed that PSCA expression was an independent prognostic factor in GC (P=0.036).

PSCA encodes a GPI-anchored cell membrane protein that belongs to the Thy-1/Ly-6 family.^6^ The protein was predicted to be in the function of cell adhesion, proliferation, and survival.^16^-^18^ Several studies have suggested that PSCA may play a role in signal transduction and cell growth regulation.^19^-^21^ Previous research have found that PSCA is highly expressed in prostate cancer, bladder cancer, renal cell cancer, pancreatic cancer, ovarian cancer and other cancer, but low in esophageal cancer and GC.^15^, ^22^, ^23^ Later studies have shown that PSCA promotes prostate cancer by increasing the expression of c-myc through the PI3K/AKT signalling pathway.^12^ A study of PSCA in esophageal cancer has shown that PSCA plays a tumour suppressor role by promoting the nuclear translocation of RB1CC1 13.

Our study indicated that PSCA suppressed cell proliferation in GC, and knockdown of PSCA gene promotes cell proliferation both *in vitro* and *in vivo*. PSCA acts a tumour suppressor role in GC, similar to the function of PSCA in esophageal cancer, but contrary to the function of PSCA in prostate cancer and nasopharyngeal cancer.^6^ All these findings demonstrated that PSCA plays different biological functions in different tissue types.^24^ PSCA functions in tumour growth are tissue-dependent.

It needs to be noted that the specific mechanism of PSCA inhibiting the proliferation of GC has not been fully explored in this study. So far, the mechanism of PSCA in GC is still unknown, PSCA plays a tumour suppressor role in GC and esophageal cancer. Whether PSCA has similar results in other gastrointestinal tumour, such as colon cancer, remains to be studied. In addition, why PSCA has different functions in different tissue types has not been fully explored at present. More studies on the expression patterns, functions, outcomes and biological mechanisms of PSCA in different tissues and cell types are needed.

## Figures and Tables

**Figure A:**
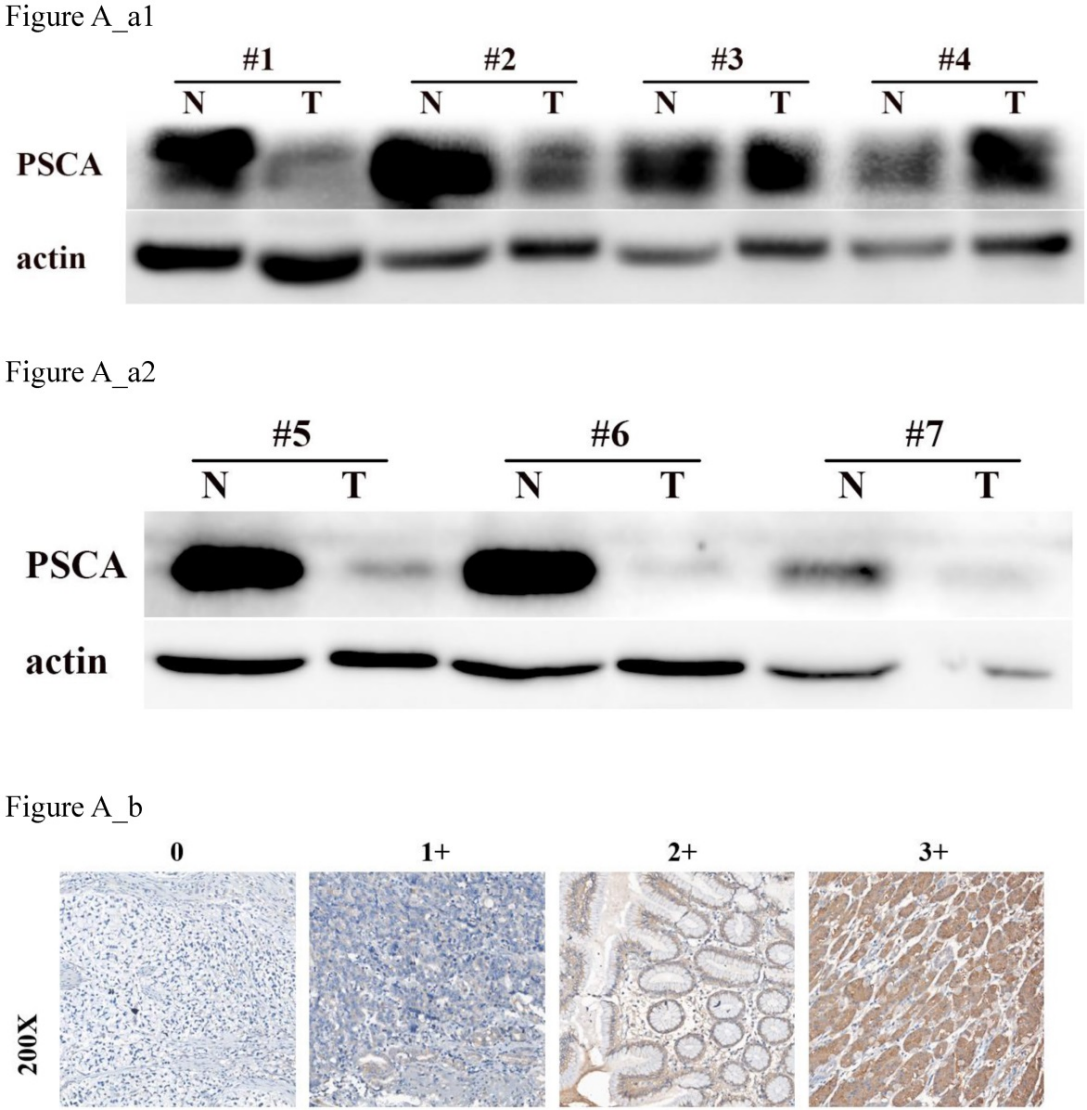
***PSCA* is lowly expressed in GC tissues.** (a) Western blot of *PSCA* expression in 7 pairs of representative GC tissues (T) with MNGT. (b) Immunohistochemical staining of *PSCA* in four pairs of representative GC tissues (T) with MNGT.

**Figure B:**
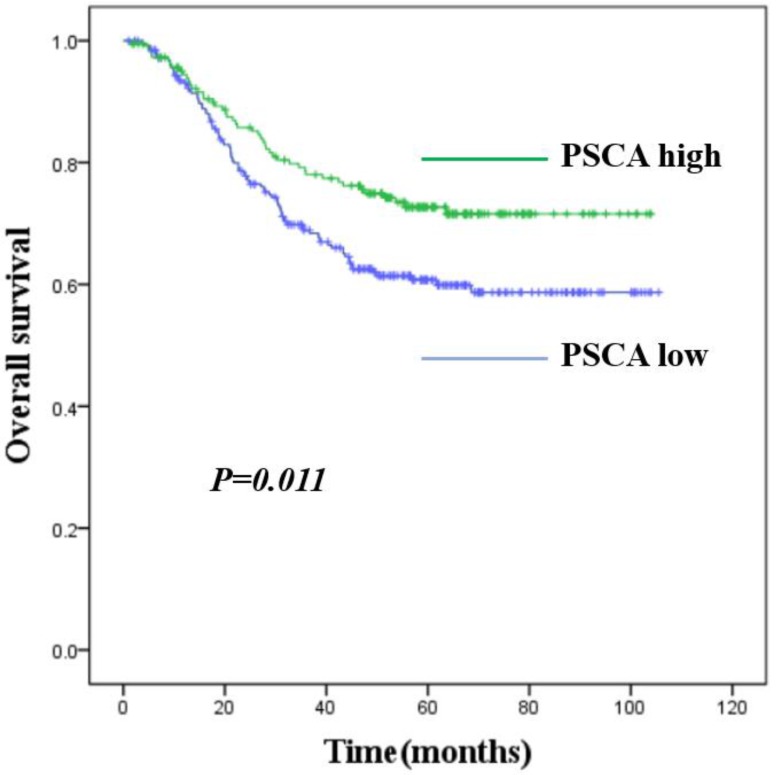
** Kaplan-Meier survival curves for GC patients with high *PSCA* expression versus low *PSCA* expression.** The overall survival of patients of GC with high or low *PSCA* expression (P=0.011).

**Figure C:**
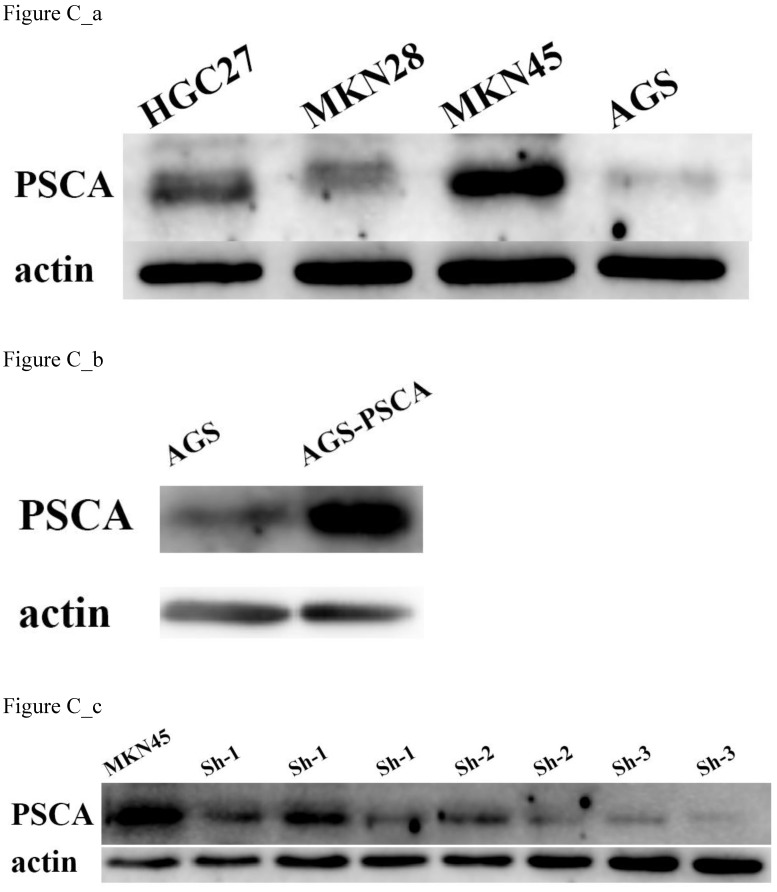
** PSCA expression in cell lines and stable cell lines construction.** (a) PSCA expression is lowest in AGS, and is highest in MKN45. (b) Overexpression of PSCA stable cell line AGS-PSCA was constructed. (c) Downregulation of PSCA stable cell line MKN45-shPSCA was constructed using the last cell line (sh-3).

**Figure D:**
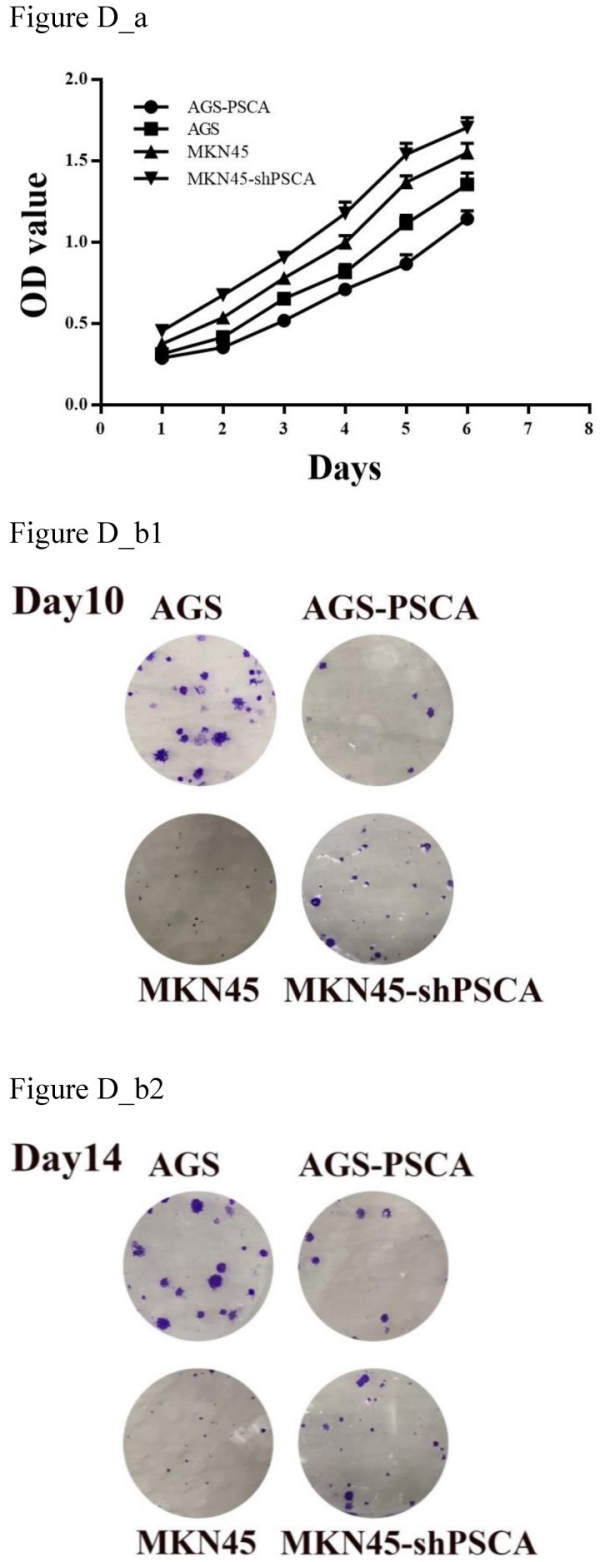
** Effects of PSCA on the cell proliferation *in vitro*.** (a) MTS assay indicated that knockdown of PSCA in MKN45-shPSCA cells increased the cell proliferation, overexpression of PSCA in AGS-PSCA cells decreased the cell proliferation. (b) Colony formation assay indicated that knockdown of PSCA in MKN45-shPSCA cells result to more and bigger colonies, overexpression of PSCA in AGS-PSCA cells result to less and smaller colonies at day10 and 14.

**Figure E:**
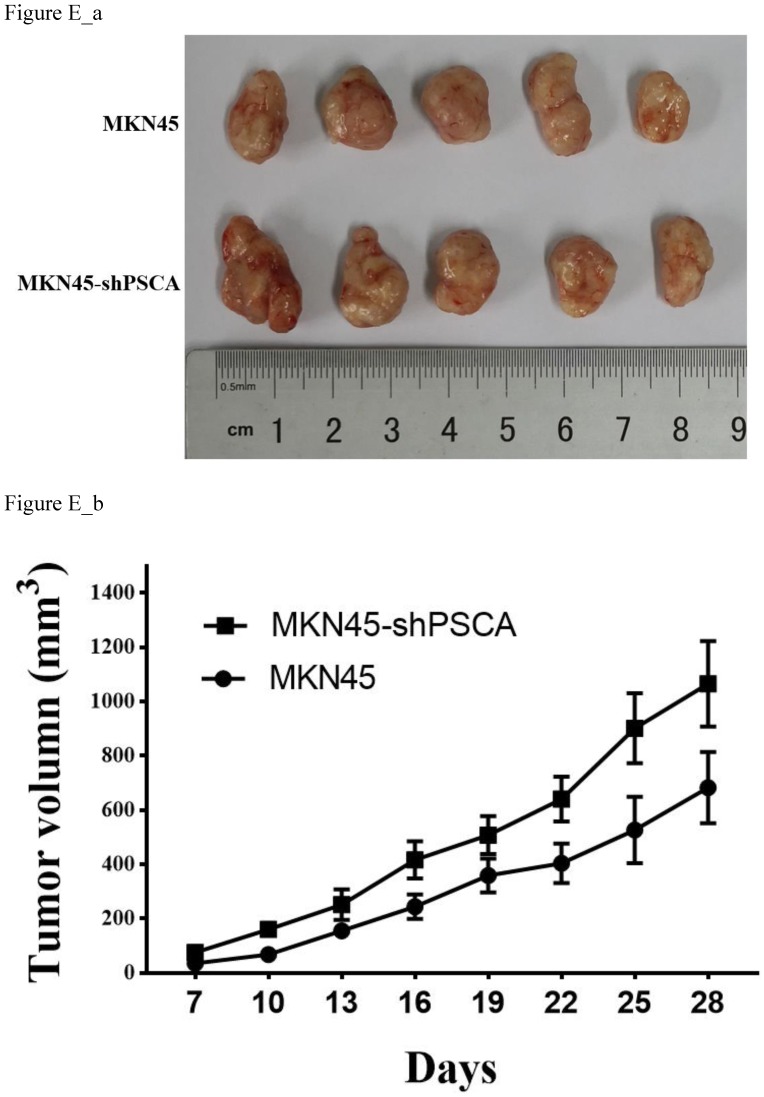
** Effects of PSCA on the cell growth *in vivo*.** (a) Tumour excised from nude mice after 4 weeks growth and then weighted. (b) Tumour volume were measured using the formula 0.5 × (length)× (width)^2^.

**Table 1:** Difference of PSCA expression between GC and MNGT using IHC staining.

Tissue type	PSCA expression		Total	P value
	low	high		
MNGT	165 (37.7%)	273 (62.3%)	438	<0.001
T	252 (57.5%)	186 (42.5%)	438	

**Table 2:** Clinicopathological characteristics and PSCA expression in 438 GC patients

Characteristics	Number of cases (%)
**Age (years)**	
<65	307(70.1)
≥65	131(29.9)
**Gender**	
Male	298(68.0)
Female	140(32.0)
**Tumour size(cm)**	
<5	280(63.9)
≥5	158(36.1)
**Histologic grade**	
Well	12(2.7)
Moderate	50(11.4)
Poor	376(85.9)
**pT classification**	
T1	67(15.3)
T2	50(11.4)
T3	153(34.9)
T4	168(38.4)
**pN classification**	
N0	140(32.0)
N1	74(16.9)
N2	77(17.6)
N3	147(33.5)
**pM classification**	
M0	406(92.7)
M1	32(7.3)
**pTNM stage**	
I	79(18.0)
II	128(29.2)
III	199(45.4)
IV	32(7.4)
**Vital status**	
Alive	300(68.5)
Death	138(31.5)
**Expression of PSCA**	
Low expression	252(57.5)
High expression	186(42.5)

**Table 3:** Correlation between PSCA expression and clinicopathological variables in 438 GC cases

Characteristics		PSCA expression		P value
		Low	High	
**Age (years)**	**<**65	176	131	0.894
	**≥**65	76	55	
**Gender**	Male	174	124	0.597
	Female	78	62	
**Tumour size (cm)**	**<**5	160	120	0.825
	**≥**5	92	66	
**Histologic grade**	Well	6	6	0.736
	Moderate	27	23	
	Poor	219	157	
**T classification**	T1	33	34	0.024*
	T2+T3	109	94	
	T4	110	58	
**N classification**	N0	69	71	0.018*
	N1	44	30	
	N2	43	34	
	N3a	81	49	
	N3b	15	2	
**M classification**	M0	232	174	0.555
	M1	20	12	
**pTNM stage**	I+II	107	100	0.019*
	III+IV	145	86	

*P-value<0.05

**Table 4:** Univariate and multivariate Cox proportional analysis with overall survival.

Parameters	Univariate analysis		Multivariate analysis	
	HR^a^ (95% CI^b^)	P	HR (95% CI)	P
**PSCA expression**	0.637 (0.448-0.906)	0.012*	0.683 (0.479-0.975)	0.036*
Low				
High				
**Age**	1.757 (1.250-2.471)	0.001**	2.007 (1.422-2.834)	<0.001**
<65				
≥65				
**Sex**	1.533(1.091-2.153)	0.014*	1.609 (1.141-2.269)	0.007*
Male				
Female				
**Tumour size (cm)**	1.802(1.288-2.521)	0.001**		0.337
<5				
≥5				
**Histologic Grade**	1.820(1.028-3.223)	0.040*		0.461
Well+moderate				
Poor				
**TNM Stage**	5.985(3.874-9.246)	<0.001**	6.053 (3.912-9.365)	<0.001**
I-II				
III-IV				
**T classification**	2.321(1.842-2.926)	<0.001**		
T1				
T2				
T3				
T4				
**N classification**	1.969(1.688-2.296)	<0.001**		
N0				
N1				
N2				
N3a				
N3b				
**M classification**	5.273(3.251-8.550)	<0.001**		
M0				
M1				

^a^HR, hazard ratio; ^b^CI, confidence interval; *P-value<0.05;**P-value<0.01
